# Effects of a low‐energy diet with and without oat bran and olive oil supplements on body mass index, blood pressure, and serum lipids in diabetic women: A randomized controlled trial

**DOI:** 10.1002/fsn3.1642

**Published:** 2020-05-20

**Authors:** Nora A. AlFaris, Adnan S. Ba‐Jaber

**Affiliations:** ^1^ Nutrition and Food Science (PhD) Department of Physical Sport Science Princess Nourah bint Abdulrahman University Riyadh Saudi Arabia; ^2^ Department of Food Science and Nutrition College of Food and Agriculture Sciences King Saud University Riyadh Saudi Arabia

**Keywords:** blood pressure, low‐energy diet, oat bran, olive oil, serum lipid, type 2 diabetes

## Abstract

Type 2 diabetes is an epidemic public health issue worldwide. It is common among adults and is more severe among overweight and obese subjects. This study was conducted to evaluate the effects of a low‐energy diet with and without oat bran and olive oil supplements on body mass index, blood pressure, and serum lipids in women with diabetes. It was conducted for three months among 78 participants after dividing them into six groups. Groups 2 to 6 were received low‐energy diet (1,600 kcal/day), with or without oat bran (10 g/day) and olive oil (5 g/day) supplements. Weight, height, blood pressure, and serum lipids were measured. A low‐energy diet with and without oat bran and olive oil supplements lowers body mass index in subjects by 0.9%–6.0% on average. It also lowers systolic and diastolic blood pressure by 1.0%–9.0% and 4.8%–12.6%, respectively. Serum triglycerides were declined in groups 2, 3, and 4 by 27.2%, 17.3%, and 1.7%, respectively, but not significantly. Total cholesterol was dropped significantly by 8.3% only when the low‐energy diet was used with oat bran supplement among obese subjects, while LDL cholesterol was dropped significantly by 20.0% only when it was used with oat bran and olive oil supplements among subjects with high serum triglycerides. Proper control of type 2 diabetes among overweight and obese adults is needed to control cardiovascular complications. This could be accomplished by following a low‐energy diet and incorporating healthy foods such as oat and olive oil into the usual diet.

## Graphical abstract

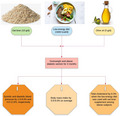

## 1 INTRODUCTION

Type 2 diabetes is the main type of diabetes that occurs mainly among adults and characterizes by elevated serum glucose caused by insulin resistance (WHO, 1999). The global prevalence of diabetes among adults in 2014 was estimated at 8.5%, which exceeds 400 million adults (WHO, 2016). Type 2 diabetes affects undesirably the patient's life quality and productivity. When diabetes is uncontrolled, the risk of premature death and several adverse health complications such as cardiovascular disease, nephropathy, visual impairment, and others, is usually raised (WHO, 1999). Cardiovascular disease risk is higher; twice to four times, in diabetic adults compared with their healthy counterparts (Nelson, Rochelau, & Nicholls, 2018). Therefore, effective treatment for diabetes should be combined with relevant management of cardiovascular disease and other complications [Gakidou et al., 2011].

Definitely, diabetic individuals need to control this disease and the associated complications via a proper mixture of diet, physical activity, and medication when needed (Garber et al., 2018). Overweight and obesity are the strongest risk factors for type 2 diabetes. Being overweight or obese increases severity of this disease and associated complications. (Vazquez, Duval, Jacobs, & Silventoinen, 2007; Zaccardi et al., 2017). Furthermore, several unhealthy dietary patterns such as over intake of total fat, saturated fat and simple carbohydrates, as well as insufficient dietary fiber consumption are connected to a higher risk of both obesity and type 2 diabetes (Ley, Hamdy, Mohan, & Hu, 2014). Interestingly, losing body weight in diabetic adults can help to control this disease severity and cardiovascular risk factors (Wing et al., 2011). In addition, a healthy diet that limits eating of saturated fatty acids and provides an adequate intake of dietary fiber help to manage type 2 diabetes and the related complications successfully (Asif, 2014). Oat bran is rich in soluble fiber, and beta‐glucans, which believed to have many beneficial effects, include lowering total and LDL cholesterol and, thus, dropping cardiovascular disease risk (Chen & Raymond, 2008). Olive oil is a rich source of monounsaturated fatty acids, specifically, oleic acid. It has proven health benefits interrelated to a lower stroke and heart disease risk (Schwingshackl & Hoffmann, 2014).

The objective of this trial was to evaluate the effects of a low‐energy diet with and without oat bran and olive oil supplements on body mass index (BMI), blood pressure, and serum lipids in women with diabetes from Saudi Arabia. We hypothesized that a low‐energy diet could improve the outcomes mentioned above in those women. Additionally, we assumed that adding oat bran and olive oil supplements could contribute to further improvement in these outcomes.

## 2 MATERIALS AND METHODS

### 2.1 Study design and subjects

**Figure 1:**
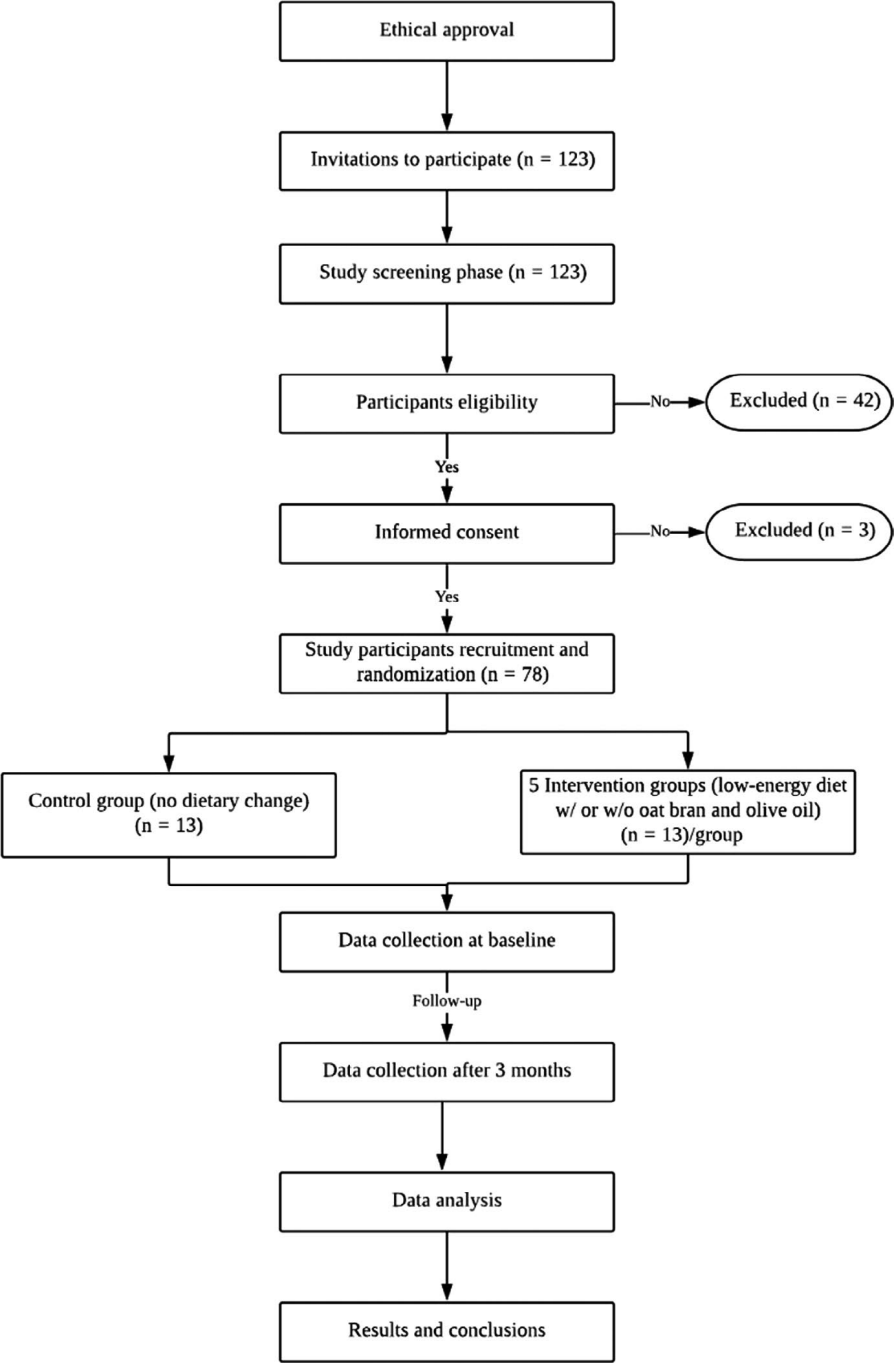
Study flowchart

This study is a three‐month randomized controlled trial with parallel design of a low‐energy diet with and without oat bran and olive oil supplements. Study flowchart is presented in Figure 1. Actually, 123 women with diabetes were invited to participate in this trial from the King Salman Hospital in Riyadh, Saudi Arabia. The systematic random sampling method was used to recruit the study subjects from women with diabetes who came for a follow‐up examination at the Center of Diabetes at King Salman Hospital in Riyadh, Saudi Arabia. The inclusion criteria were Saudi nonpregnant or lactating women, aged 25–60 years, had overweight or obesity (BMI = 25–29.9 or ≥ 30, respectively), diagnosed with type 2 diabetes, do not suffer from typical diabetes symptoms, and do not use insulin replacement therapy. In fact, only 78 women were willing to participate in and complete this study. Participants were randomized into six groups of 13 subjects in each group by using a purposive sampling method. Randomization was performed by using block randomization in three consecutive steps. In the first step, subjects in groups 3 and 4 were chosen randomly from subjects characterized by high serum triglycerides (˃2.3 mmol/L). In the second step, subjects in groups 5 and 6 were chosen randomly from obese subjects. Finally, the remaining subjects were assigned randomly either to group 1 or group 2. Written informed consent was obtained from all study participants in accordance with Helsinki Declaration. This study was approved by the Institutional Review Board of King Salman Hospital, Riyadh, Saudi Arabia.

### 2.2 Dietary interventions

Different groups of subjects were received different dietary interventions as following. Group 1 subjects did not receive any nutrition education, meal plans, or any supplements (control group). On the other hand, subjects in groups 2 to 6 were received nutrition education and meal plans for low‐energy diet, with or without oat bran and olive oil supplements. Nutrition education and meal plans were given by professional dietitians from the Center of Diabetes at King Salman Hospital in Riyadh, Saudi Arabia at the study baseline and the follow‐up visits. Nutrition education was designed to clarify the critical role for dietary management in control of type 2 diabetes complications. Meal plans for low‐energy diet were prepared based on the participants’ health status and food preferences. Daily meal plans of the low‐energy diet were composed of five balanced meals (three main meals and two snacks) and provide 1,600 kcal per day; where 50%–60%, 20%–30%, and 12%–20% of daily energy derives from carbohydrate, fat, and protein, respectively. As well as, high‐fiber carbohydrate sources were used.

In addition to the low‐energy diet, subjects in group 2 were not given any supplements. However, subjects in groups 3 and 5 were given only 10 grams daily of oat bran supplement, while those in groups 4 and 6 were given supplements of oat bran (10 grams per day) and olive oil (5 grams per day). Oat bran supplement was given to subjects in the form of tablets (Good ‘n’ Natural Manufacturing, New York, USA). The subjects were requested to take them 15 min before breakfast, lunch, and dinner (3, 4, and 3 grams per day, respectively). Extra virgin olive oil was collected from a local natural products store. The participants were requested to add 5 g of olive oil to salad at lunch daily. The participants were contacted regularly during the study (once every two weeks at least) to follow‐up on the trial progress. The food intake of participants was evaluated by using 24‐hr recall when they contacted or during the follow‐up visits to ensure compliance with the study’ dietary interventions.

### 2.3 Data collection

The diagnosis of study participants with type 2 diabetes was confirmed from their medical files. Data were collected from participants directly at baseline and the end of the trial (3 months later). Collected data include socio‐demographic characteristics, height, weight, systolic and diastolic blood pressure, and biochemical analysis of serum lipids. Body height was measured to closest 1 millimeter, and body weight was measured to closest 100 grams using standardized methods. Body mass index (BMI) was obtained by dividing weight in kilogram over height squared in meter. Systolic blood pressure and diastolic blood pressure were measured by a registered nurse by using a manual sphygmomanometer (Diplomat‐presameter, Riester, Germany).

After overnight fasting, blood samples were collected from all study subjects in clinical laboratory of King Salman Hospital. Plasma was prepared by centrifugation (3,500 rpm, 3 min). Biochemical analysis of serum lipids, which includes triglycerides, total cholesterol, LDL cholesterol, and HDL cholesterol, was performed by using standardized methods.

### 2.4 Statistical analysis

SPSS version 23 was used for data analysis. Categorical variables and continuous variables were presented as percentages and means (±*SD*), respectively. Noticed changes in continuous variables were also expressed as percentages. Paired samples *t* test was used to test the significance of differences between pretest and post‐test values for each studied outcome within each group. Moreover, one‐way ANOVA test was used to test statistically significance of differences between the means of the noticed change values for each studied outcome within different groups. *p* values were completed assuming two‐tailed tests. Differences were seen statistically significant when *p* values < .05.

## 3 RESULTS

Overall, 78 women with diabetes have participated in this trial. Socio‐demographic characteristics of study subjects, including age‐groups, education level, marital status, and monthly family income, were presented in Table 1.

**Table 1:** Socio‐demographic characteristics of study subjects

Variables	Study Subjects (%)
Age Groups	
25–39 years	26.0%
40–50 years	37.4%
51–60 years	36.6%
Education Level	
Illiterate	74.0%
High school education or less	23.6%
College education or more	2.4%
Marital Status	
Single	4.9%
Married	69.1%
Divorced/ widowed	26.0%
Monthly Family Income^a^	
5,000 SR or less	62.6%
5001–10000 SR	25.2%
More than 1,000 SR	12.2%

^a^ SR is Saudi Riyal (1 USD ≅ 3.75 SR).

Table 2 presented the means of BMI, blood pressure, and serum lipids of the subjects at baseline (pretest values) and end of the three‐month trial (post‐test values) of a low‐energy diet with and without oat bran and olive oil supplements and the noticed change in the studied outcomes. Subjects’ average BMI at baseline ranged from 29.3 (group 3) to 38.9 (group 6). Data revealed that the average BMI for control group was elevated at end of this trial by 0.2; thus, BMI was increased by 0.6% on average. Contrarily, BMI means for the five intervention groups were dropped at the end of this trial by a value ranged from 0.3 to 2.2. Actually, BMI was declined by 3.2% on average for all subjects from the five intervention groups; where the reduction in BMI was 3.8%, 1.7%, 0.9%, 6.0%, and 3.6% for groups 2 to 6, respectively. The BMI means of pretest and post‐test values were significantly different only in the case of groups 2 and 6. Furthermore, the mean for noticed change in BMI for group 6 was significantly different from that for the control group.

**Table 2:** Body mass index (BMI), blood pressure, and serum lipid profile of subjects at baseline (pretest) and end of three‐month trial (post‐test) of low‐energy diet with and without oat bran and olive oil supplements (means (*SD*), and the noticed change between pretest and post‐test values (means (*SD*, %))

Variables	Study Groups of Subjects						*p* value
	Group 1 (Control)	Group 2 (LED)	Group 3^a^ (LED & OB)	Group 4^a^ (LED, OB & OO)	Group 5^b^ (LED & OB)	Group 6^b^ (LED, OB & OO)^c^	
BMI (kg/m^2^)							
Pretest	36.3 (7.8)	36.7 (9.5)	29.3 (2.7)	32.9 (7.5)	36.7 (7.6)	38.9 (7.0)	.019**
Post‐test	36.5 (8.0)	35.3 (9.1)*	28.9 (2.3)	32.6 (7.1)	34.5 (6.1)	37.5 (6.2)*	
Noticed change	0.2 (1.3, 0.6)^a^	−1.4 (1.4, −3.8)^a^, ^b^	−0.5 (1.1, −1.7)^a^, ^b^	−0.3 (1.0, −0.9)^a^, ^b^	−2.2 (4.9, −6.0)^a^, ^b^	−1.4 (1.2, −3.6)^b^	
Systolic blood pressure (mmHg)							
Pretest	113.1 (13.8)	120.0 (115.3)	125.4 (17.1)	123.8 (16.6)	132.7 (23.9)	123.8 (12.6)	.367
Post‐test	115.4 (20.3)	118.8 (10.4)	120.8 (15.5)	120.0 (16.3)	120.8 (16.6)*	121.5 (14.6)	
Noticed change	2.3 (15.9, 2.0)	−1.2 (15.3, −1.0)	−4.6 (12.0, −3.7)	−3.8 (21.0, −3.1)	−11.9 (12.5, −9.0)	−2.3 (19.2, −1.9)	
Diastolic blood pressure (mmHg)							
Pretest	80.0 (7.9)	84.2 (8.6)	78.5 (6.9)	81.9 (10.3)	85.8 (12.9)	85.8 (7.0)	.137
Post‐test	77.3 (9.7)	76.5 (6.3)*	74.6 (6.6)	77.7 (9.0)	75.0 (9.1)*	75.8 (5.7)*	
Noticed change	−2.7 (13.2, −3.4)	−7.7 (7.0, −9.1)	−3.8 (8.7, −4.8)	−4.2 (7.0, −5.1)	−10.8 (7.6, −12.6)	−10.0 (11.0, −11.7)	
Serum triglycerides (mmol/L)							
Pretest	2.38 (1.03)	1.73 (0.97)	3.65 (1.47)	3.51 (1.69)	1.58 (0.45)	1.55 (0.28)	.048**
Post‐test	4.31 (4.72)	1.26 (0.35)	3.02 (0.93)	3.45 (1.44)	1.72 (0.68)	1.68 (0.51)	
Noticed change	1.93 (4.27, 81.1)^a^	−0.47 (0.84, −27.2)^b^	−0.63 (1.06, −17.3)^b^	−0.06 (0.93, −1.7)^a^, ^b^	0.15 (0.54, 9.5)^a^, ^b^	0.14 (0.44, 9.0)^a^, ^b^	
Serum total cholesterol (mmol/L)							
Pretest	5.88 (0.97)	5.63 (1.07)	5.00 (1.68)	6.31 (1.49)	5.20 (1.03)	5.32 (1.01)	.189
Post‐test	6.14 (0.79)	5.25 (1.02)	5.25 (0.94)	5.72 (0.91)	4.77 (0.85)*	5.11 (1.09)	
Noticed change	0.25 (0.94, 4.3)	−0.38 (0.66, −6.7)	0.25 (1.82, 5.0)	−0.59 (1.17, −9.4)	−0.43 (0.61, −8.3)	−0.22 (0.45, −4.1)	
Serum LDL cholesterol (mmol/L)							
Pretest	3.91 (1.13)	3.85 (0.93)	2.17 (2.12)	3.75 (1.36)	3.37 (1.01)	3.46 (0.88)	.183
Post‐test	2.97 (2.17)	3.72 (0.88)	2.83 (1.03)	2.99 (1.23)*	2.97 (0.83)	3.18 (1.04)	
Noticed change	−0.92 (1.72, −23.5)	−0.13 (0.54, −3.4)	0.67 (1.98, 30.9)	−0.75 (1.05, −20.0)	−0.40 (0.67, −11.9)	−0.28 (0.55, −8.1)	
Serum HDL cholesterol (mmol/L)							
Pretest	0.91 (0.48)	0.99 (0.24)	1.17 (0.34)	0.98 (0.29)	1.11 (0.42)	1.16 (0.34)	.069
Post‐test	1.21 (0.26)	0.96 (0.31)	1.04 (0.25)	1.16 (0.49)	1.02 (0.25)	1.15 (0.22)	
Noticed change	0.30 (0.50, 33.0)	−0.03 (0.32, −3.0)	−0.13 (0.33, −11.1)	0.18 (0.41, 18.4)	−0.09 (0.44, −8.1)	−0.01 (0.43, −0.9)	

^a^ Subjects with high serum triglycerides (˃2.3 mmol/L).

^b^ Obese subjects (BMI ≥ 30)

^c^ LED means low‐energy diet, OB means oat bran supplement (10 g/day), and OO means olive oil supplement (5 g/day).

* A significant difference between pretest and post‐test values was found by using paired samples *t* test (*p* ˂.05).

** A significant difference between means of the noticed change among different groups was found by using one‐way ANOVA test. Values with different letters, within a row, are significantly different (*p* ˂.05).

Mean systolic blood pressure for participants at baseline was ranged from 113.1 mmHg (group 1) to 132.7 mmHg (group 5), whereas mean diastolic blood pressure at baseline was ranged from 78.5 mmHg (group 3) to 85.8 mmHg (groups 5 and 6). Average blood pressure values for control group at end of this trial have exhibited a rise in the systolic blood pressure by 2.3 mmHg (2.0%) and a drop in the diastolic blood pressure by 2.7 mmHg (3.4%). The mean systolic and diastolic blood pressure values for all five intervention groups were dropped at the end of this trial by a value ranged from 1.2 to 11.9 mmHg (1.0%–9.0%) and 3.8 to 10.8 mmHg (4.8%–12.6%), respectively. Furthermore, means of pretest and post‐test values were significantly different in group 5 only for the systolic blood pressure and in groups 2, 5, and 6 for diastolic blood pressure. However, the means for noticed change in both systolic and diastolic blood pressure among different groups did not differ significantly.

Serum lipids include four basic parameters: serum triglycerides, total cholesterol, LDL cholesterol, and HDL cholesterol. Mean serum triglycerides for participants at baseline were ranged from 1.55 mmol/L (group 6) to 3.65 mmol/L (group 3). Average serum triglycerides values raised at the end of trial in groups 1, 5, and 6 by 1.93 mmol/L (81.1%), 0.15 mmol/L (9.5%), and 0.14 mmol/L (9.0%), respectively, and dropped in groups 2, 3, and 4 by 0.47 mmol/L (27.2%), 0.63 mmol/L (17.3%), and 0.06 mmol/L (1.7%), respectively. There was no significant difference between means of pretest and post‐test values. However, the means for noticed change in serum triglycerides for groups 2 and 3 were significantly different from that for the control group. Average serum total cholesterol, LDL cholesterol, and HDL cholesterol for participants at baseline were ranged from 5.00 to 6.31 mmol/L, 2.17 to 3.91 mmol/L, and 0.91 to 1.17 mmol/L, respectively. Furthermore, means of pretest and post‐test values were significantly different only in group 5 for the serum total cholesterol and group 4 for the serum LDL cholesterol. However, the means for noticed change in serum total cholesterol, LDL cholesterol, and HDL cholesterol were not significantly different among different groups.

## 4 DISCUSSION

Present trial demonstrated that three months of low‐energy diet (1,600 kcal/day) with and without oat bran and olive oil supplements confer wide‐ranging health benefits in women with diabetes. Based on the current results, a low‐energy diet with and without oat bran and olive oil supplements was capable of lowering BMI on average by 3.2% (0.9%–6.0%). However, a significant decline in BMI was seen only with a low‐energy diet without supplements (3.8%) and low‐energy diet with oat bran and olive oil supplements when used with obese women (3.6%). Our results were consistent with the currently available data. A recent systematic review quantifies the effectiveness of low‐energy diets in achieving weight loss in diabetic adults. They reported predicted changes in body weight and BMI for different energy‐restricted diets (400–1,600 kcal/day) during different time intervals (2 weeks to 4 months). At three‐month interventions, there was a BMI decrease of 2.3% (0.4%–4.3%) observed on a low‐energy diet of 1,600 kcal per day (Kloecker et al., 2019). Furthermore, the mean for noticed change in BMI for obese women followed a low‐energy diet with oat bran and olive oil supplements (group 6) was significantly different from that for the control group. Oat bran is rich in soluble fiber and beta‐glucans, which may help to suppress hunger hormones such as ghrelin and boost fullness hormones such as cholecystokinin, and thus, may support weight loss (Beck, Tosh, Batterham, Tapsell, & Huang, 2009). Furthermore, there is evidence supporting a lower prevalence of obesity in adults who consume olive oil regularly (Soriguer et al., 2009).

Results of current trial showed that a low‐energy diet with and without oat bran and olive oil supplements could lower systolic and diastolic blood pressure in women with diabetes. However, the low‐energy diet was successful in lowering systolic and diastolic blood pressure significantly by 9.0% and 12.6%, respectively, when used with oat bran supplement among obese women. Many studies reported that oat bran and its beta‐glucans could significantly lower both systolic and diastolic blood pressure either for healthy adults or adults with pre‐existing high blood pressure (Evans et al., 2015; Keenan, Pins, Frazel, Moran, & Turnquist, 2002). Moreover, the low‐energy diet was effective in lowering diastolic blood pressure significantly by 11.7% when used with oat bran and olive oil supplements among obese women. There is growing evidence to suggest that olive oil and particularly oleic acid could be with a potential to reduce blood pressure. High intake of olive oil is proposed to raise membranes’ oleic acid content, which help to manage membrane lipid structure and produce a decline in blood pressure (Terés et al., 2008).

Our results showed diverse effects of a low‐energy diet with and without oat bran and olive oil supplements on serum lipids in women with diabetes. Serum triglycerides were notably elevated in the control group by 81.1% and decline in groups 2 (27.2%), 3 (17.3%), and 4 (1.7%), but not significantly. We inferred that the low‐energy diet was able to lower serum triglycerides when used without supplements and when used with supplements for women with high serum triglycerides. The noticed change was statistically significant compared with control group when the low‐energy diet was used without supplements and with oat bran supplement among subjects with high serum triglycerides. In addition, the total cholesterol was dropped significantly by 8.3% only when the low‐energy diet was used with oat bran supplement among obese subjects. While LDL cholesterol was dropped significantly by 20.0% only when it was used with oat bran and olive oil supplements among subjects with high serum triglycerides. Oat bran contains beta‐glucans a soluble fiber that has been proven to lower serum cholesterol. Beta‐glucans could be lower serum cholesterol due to their ability to remove cholesterol‐rich bile, a substance that facilitates fat digestion and absorption (Sima, Vannucci, & Vetvicka, 2018]. In a review of randomized controlled trials, daily eating at least three grams of oat beta‐glucan was able to reduce total and LDL cholesterol by 0.3 mmol/L and 0.25 mmol/L, respectively. However, there is no significant effect observed of oat beta‐glucan on serum triglycerides or HDL cholesterol. An interesting finding from this review is that the lowering effect on total and LDL cholesterol was significantly greater in diabetic adults compared with their counterpart adults without diabetes (Whitehead, Beck, Tosh, & Wolever, 2014). Many studies demonstrate that persons when consumed olive oil regularly have a much lower cardiovascular disease risk (Martinez‐Gonzalez, Dominguez, & Delgado‐Rodríguez, 2014; Schwingshackl & Hoffmann, 2014). Besides high monounsaturated fatty acid level, olive oil contains phenolic compounds which exhibit antioxidant and anti‐inflammatory activities. Consequently, it helps in preventing lipoperoxidation and carrying out favorable changes in serum lipid profile, thus reducing cardiovascular risk (Pérez‐Martínez, García‐Ríos, Delgado‐Lista, Pérez‐Jiménez, & López‐Miranda, 2011).

Current evidence emphasized on the importance of early and comprehensive treatment for type 2 diabetes to enable those patients to have long‐term healthy lives. The treatment should consider reducing cardiovascular risk factors, mainly lowering of blood pressure and serum lipids, and consequently preventing progress of cardiovascular disease in diabetic individuals (Herman et al., 2015). Patients with diabetes need counseling on a healthy diet to promote healthier lifestyles. Current guidelines of medical nutrition therapy for type 2 diabetes focus on implementing a lower energy intake for people with overweight or obesity, avoiding free sugars, increasing intake of dietary fiber, and replacing saturated fats with mono‐ and polyunsaturated fats (American Diabetes Association, 2019; Evert et al., 2019). Therefore, relevant cultural and environmental strategies are needed to encourage and aid diabetic individuals to reach healthy body weight and dietary patterns (Cradock et al., 2017). Generally, current evidence suggests that a body weight loss of 5% or more in diabetic adults will provide widespread health benefits; mainly lower cardiovascular risk and better life quality (Franz, Boucher, Rutten‐Ramos, & VanWormer, 2015; Strelitz et al., 2019; Wilding, 2014). It has been proposed that energy restriction through a low‐energy diet can aid to lose weight and contribute to the reduction of cardiovascular complications and reversal of hyperglycemia related to type 2 diabetes when weight loss is maintained (Bynoe, 2016; Strelitz et al., 2019). Furthermore, it may be beneficial to include plenty of healthy foods such as oat bran and olive oil in the usual daily diet for those with type 2 diabetes (Chen & Raymond, 2008; Schwingshackl & Hoffmann, 2014).

The study had a few limitations. The first limitation is the relatively small sample size due to time and logistic constrains. Second, the physical activity of participants was not assessed in this study. However, this study still affords valuable data about the effects of a low‐energy diet with and without oat bran and olive oil supplements on BMI, blood pressure, and serum lipids in women with diabetes.

In conclusion, three months of low‐energy diet provide 1,600 kcal daily with and without oat bran and olive oil supplements may carry out health benefits for overweight and obese diabetic women. Overweight and obesity are key modifiable risk factors for type 2 diabetes as well as its complications. Therefore, losing weight may help to lower cardiovascular complications connected to type 2 diabetes.

## CONFLICT OF INTEREST

The authors declare that they do not have any conflict of interest.

## ETHICAL APPROVAL

This study was approved by the Institutional Review Board of King Salman Hospital, Riyadh, Saudi Arabia.

## INFORMED CONSENT

Written informed consent was obtained from all study participants.
